# A theory of scaling for community-based fisheries management

**DOI:** 10.1007/s13280-021-01563-5

**Published:** 2021-06-03

**Authors:** Dirk J. Steenbergen, Andrew M. Song, Neil Andrew

**Affiliations:** 1 Australian Centre for Ocean Resources and Security (ANCORS), University of Wollongong (UOW), North Wollongong, NSW 2500 Australia; 2 Faculty of Arts and Social Sciences, University of Technology Sydney, Ultimo, NSW 2007 Australia

**Keywords:** Community-based fisheries management, Collective action, Innovation, Practice-oriented multi-level perspective on innovation and scaling (PROMIS), Scaling

## Abstract

Community-based approaches to fisheries management has emerged as a mainstream strategy to govern dispersed, diverse and dynamic small scale fisheries. However, amplifying local community led sustainability outcomes remains an enduring challenge. We seek to fill a theoretical gap in the conceptualization of ‘scaling up community-based fisheries management’. We draw on literature of agriculture innovations to provide a framework that takes into account process-driven and structural change occurring across multiple levels of governance, as well as different phases of scaling. We hypothesize that successful scaling requires engagement with all aspects of a governing regime, coalescing a range of actors, and therefore, is an enterprise that is larger than its parts. To demonstrate where the framework offers value, we illustrate the development of community-based fisheries management in Vanuatu according to the framework’s main scaling dimensions.

## Introduction

With less than a decade to run, there is increasing international focus on how governments are tracking toward achieving the Sustainable Development Goals (SDGs). The 17 interconnected goals boldly state ambitions to address global challenges in food security and human development. There have been mixed results thus far against the 169 SDG targets and with that, a growing urgency among donors and international agencies to accelerate progress. Much of this ambition and imperative to move from ‘small and few’ to ‘large and many’ has been captured in the need to have impact at scale. Scaling has become integral to the vocabulary of rural development and sustainability programming (e.g. Gargani and McLean 2017; Butler et al. 2020; Lam et al. 2020; Sartas et al. 2020). ‘Scaling up’ often features prominently in programme design and theories of change (ToC), wherein successful ideas or practices need to be ‘brought to scale’. In moving from the activities, outputs, and intermediate outcomes that typically populate the earlier stages of ToC to the final outcomes and impacts needed to effect on-ground change, attribution becomes muddier and accountabilities less clear.

More profoundly, scaling has become a critical research frontier that requires new transdisciplinary methods, relationships and modes of working. The literature on scaling has flourished in recent years as researchers and development practitioners grapple with the complexities and accountabilities of scaling. The subset of this literature most relevant here has focused on scaling agricultural innovations (e.g. Cooley and Linn 2014; Hermans et al. 2017; Dror 2020; Schut et al. 2020) and on the creation of ‘innovation platforms’ and exchanges to promote widespread uptake of successful ideas (Seifu et al. 2020).

Despite this attention, ‘scaling’ remains a stubborn problem; transformative change is not the norm and development trajectories are too seldomly overturned. Critiques of the scaling literature suggest it remains too dependent on a theoretical framing provided by the diffusion of innovation literature (Rogers 2003; Johnson and Hagström 2005) with its focus on technologies and the individual behaviours of innovators and adopters. This framing, it is thought, does not adequately account for governance contexts and cultural dimensions of how people live their lives and manage risk, and relies on the modality of spreading ‘success’ from case studies, proof of concept pilots, or nodes of learning (Lam et al. 2020). At worst, these framings situate innovation separately from the socio-political environment in which adoption and spreading take place.

### Community-based fisheries management

Here, we are concerned with scaling community-based fisheries management (CBFM). Although CBFM has emerged in many guises (Aswani et al. 2012), broad principles remain the same; namely that the resulting management is enacted by and for communities and that collective action towards the management of shared resources (sensu Wade 1987) encompasses ecosystem and social dimensions, and not just the sustainability of harvests.

With the ‘tropical majority’ (Kurien 2002) highly dependent on fish for food and income, the need for widespread sustainable management of inshore fisheries has never been more necessary (Aswani et al. 2012; Batista et al. 2014). Physical and political remoteness of many coastal fishing communities in this part of the world, combined with often scarce state resources, means that national agencies are limited in their ability to centrally govern the dispersed, diverse and dynamic context of small-scale fisheries. In response to this limitation, CBFM has become a mainstream strategy in national programs for co-management of nearshore fisheries (Evans et al. 2011). This is evidenced in, for example, how CBFM forms a central component of high-level visions agreed globally for small-scale fisheries, as referenced in the 2015 Voluntary Guidelines for Securing Small-Scale Fisheries (FAO 2015). Much of the work by government agencies, together with the support of non-governmental organizations (NGO) and academic communities, has, therefore, focused on finding effective ways to introduce, strengthen or support collaborative management arrangements by funding, researching and institutionalizing CBFM initiatives around the world (Wilson et al. 2003; d’Armengol et al. 2018).

### Scaling CBFM

The question of how to amplify community-driven sustainability outcomes beyond a selected number of ‘pilot communities’ looms large. Despite significant progress in learning what forms of CBFM work in small places, a gap persists in understanding how spread of management capacity across broader constituencies can be achieved. Jagers et al. (2019) argue that spreading collective action institutions across larger domains requires deliberate and consistent intervention by external (third) parties such as governments, multilateral organisations, and even businesses.

In the context of small-scale fisheries, attempts to bring CBFM to a wider coverage have been implemented in various locales with examples including Locally Managed Marine Areas (LMMAs) in the Asia–Pacific (Govan 2009; Steenbergen and Warren 2018), inshore fisheries management based on Territorial Use Rights for Fisheries (TURFs) in Belize (Fujita et al. 2017), Chile (Gelcich et al. 2017) and South Korea (Song 2015), and ecosystem-based management in Japan (Makino et al. 2009) and the Philippines (Eisma-Osorio et al. 2009; Lowry et al. 2009). However, most scaling efforts have generated mixed results with adoption slowing once a saturation point is reached and/or reversing when initial excitement and momentum fades (Mascia and Mills 2018; Mills et al. 2019).

Much of the theory informing scaling initiatives continues to rely on perspectives that treat CBFM innovations as something that is replicable and adoptable in a relatively similar form across many contexts. We argue that this framing struggles to capture the essential attributes of CBFM, where the agency, modus operandi and social-cultural-political function of community institutions have been de-emphasized. Hence, rather than technical prescriptions, it might be more accurate to consider CBFM as a set of principles around collective action and sustainability that incorporate notions of social justice, stewardship, fairness, equity, leadership, and conflict resolution. Further, scaling these principles has to be framed more broadly than just communities, individual projects or individual national agencies.

Model-based approaches seeking to ‘roll out’ CBFM, moreover, pay insufficient attention to what ‘scaling’ means for the communities expected to implement it, and about how the principles of CBFM may differ or clash with the norms under which communities operate. Such approaches require a refocus that integrates understandings of broader institutional and specific contextual conditions, and their conduciveness to catalyse spread.

Considering the unique challenges associated with scaling a bundle of principles, the objective of this paper is to develop a conceptual framework that better captures complexities of scaling CBFM. The framework is grounded in the diverse literature on scaling that includes related ideas such as governance transformations and societal level transitions. Specifically, we utilise the ‘PRactice-Oriented Multi-level perspective on Innovation and Scaling’ (PROMIS) framework developed in the context of scaling agricultural innovations (Wigboldus et al. 2016, 2017). In doing so, we take up Wigboldus et al. (2016)’s invitation to apply perspectives from PROMIS across new sectors.

## Conceptual underpinnings of a theory of scaling

Before discussing a theory of scaling for CBFM we outline below a series of considerations that seek to go beyond the conventional calls to create ‘innovation platforms’, or ‘diffusion environments’. In doing so, we unpack drivers, influential processes, repercussions, and unforeseen impacts during scaling initiatives.

The PROMIS framework conceptualizes scaling processes as an integral part of a systematic approach to innovation, and, therefore, not something to be taken for granted (i.e. ‘once we have innovated, it will then naturally go to scale’). Analytically, it combines two approaches. First, the multi-level perspective (MLP) (Geels 2019) provides a lens through which to understand how innovations ‘travel’ across multi-scalar structures to ultimately be absorbed into systems and practice; moving from small experimental innovation to institutionalisation to broad societal absorption. Secondly, modal aspect theory is applied as a means to understand change pathways that result from scaling initiatives (Wigboldus et al. 2016). It helps in revealing the diversity and coherence (or lack thereof) of everyday things and events, and ensures a broad recognition of how scaling can affect different aspects of people’s lives or society as a whole. We focus particularly on the contributions of the multi-level perspective, given the already strong body of work in fisheries social science that explores the various impacts of governing initiatives on the complex lives and livelihoods of small-scale fishers (e.g. Jentoft and Chuenpagdee 2015), which the modal aspect dimension of PROMIS addresses.

In the following sections we elaborate on three important considerations for when new ideas and/or practices are introduced into existing regimes, which are derived from the PROMIS framing around scaling agriculture innovations (Wigboldus et al. 2016). This roots our framework in, firstly, a reflective perspective that considers possible consequences and implications of scaling; secondly, an understanding that deliberate innovations enter regimes through either direct change interventions (push) or measures that incentivise behaviour change (pull); and thirdly, a recognition that people and institutions follow habituated patterns that over time has become accepted practice and influence if and how new innovations are taken up.

### Responsible scaling

‘Responsible innovation and scaling’ requires that negative effects of scaling be anticipated (Wigboldus and Leeuwis 2013). This is a significant departure from a conventional understanding of scaling which assumes scaling as an inherently positive endeavour (i.e. more of ‘good’ things everywhere equals progress). Responsible scaling acts upon the admission that what is promoted as a solution and scaled at one point in time may later be considered a hazard; that technologies and practices working in a particular geographical or sociocultural domain may not work in other areas (and may even be counterproductive); that while some people reap the benefit from the scaled up innovation, others may be disadvantaged by it; and that when something has gone to scale, it may be difficult to reverse, even in the face of negative side effects (see also ‘moral justification of scaling’ in Gargani and McLean 2017; Augenstein et al. 2020).

Considering responsible scaling requires seeing scaling as a part of a more continuous, cumulative process that is subject to ongoing fine-tuning (or even a wholesale correction when appropriate). This counters the conventional treatment of scaling as a one-off activity by which enabling conditions for scaling such as local adaptation processes, conducive institutional and market environments, or diffusion mechanisms are established. Also, by recognizing our insufficient capacity for predicting the long-term impact of scaled innovations, responsible scaling calls for making the purpose orientation of scaling explicit—what, for who, and why—, and then adjusting expectations and strategies as scaling practices take shape. Most fundamentally, the goal of scaling may require transitioning from developing a model to be replicated, to instead achieving a possible means to empower intended beneficiaries or benefit society (Muilerman et al. 2018).

### Push and pull approaches to introducing innovations

As with the common ‘carrot and stick’ analogy (Leeuwis 2000), adoption of innovation occurs by means of ‘push’ and ‘pull’ (Wigboldus et al. 2016; Geels 2019; Totin et al. 2020). Push approaches involve the deliberate introduction of something new to a system, like a technology or model of practice. A technology-focused approach promotes a technology that addresses a development problem as the subject-to-be-scaled. Such application is often justified by theories of change that assume ‘if we introduce this, then that problem is solved’. In doing so, less attention is given to broader impacts beyond the defined problem–solution pairing. Goudzwaard et al. (2007) argue that technical solutions can result in new issues or even intensification issues as a consequence of narrow framing (see also ‘solution paradox’). Model-focused approaches seek to overcome the complexity, variability and dynamism of contexts across large spaces (Wigboldus et al. 2016) by putting forward sets of defined processes and concepts as the subject for scaling. However, development experience has shown all too often how model approaches can produce blueprint solutions (Aswani et al. 2017), reinforce uneven power relations (Béné et al. 2009), and create self-fulfilling outcomes (Steenbergen et al. 2017).

Pull approaches on the other hand develop from a demand by a governance regime to deal with a persistent or new problem. Pull approaches may also be deliberate, like when demands are produced that require a response or solution, even though that solution may yet to be defined. Such approaches therefore seek to develop conducive systems and mechanisms that allow influential actors to mobilize into innovating solutions that ultimately lead to desired things happening, like behaviour change. Most commonly, innovations in the agriculture and development sector have drawn on push approaches, relying on new technology or models to drive change in practice, without sufficient application of pull (or the combination of both) approaches to social change (Wigboldus et al. 2016).

### Path dependency

Path dependency refers to the way a regime routinely operates, contributing to perceived stability. Core institutional beliefs, values, practices and rules are difficult to change. They arise from repetitive processes that have evolved to work in certain ways for certain reasons. Over time actors develop vested interest in the way things operate, interdependencies among actors or organizations develop that cement power relations and hierarchy. This may further translate to resistance for change or innovation in a regime, effectively reinforcing path dependency. Disturbances to a regime, however, can rearrange, even if just temporarily, its institutional make-up, allowing a window of opportunity for the regime to take up an innovation and deviate from its path dependence (Tongur and Engwall 2017). Uptake of innovations does not rid a regime of path dependency per se, but rather shifts or creates new path dependencies. Innovations therefore should be understood to be putting scaling processes onto a path dependent course, which on one hand is necessary to guide desired development but on the other hand can limit creative options that will see it deviate from status quo (Muilerman et al. 2018).

## A Framework for scaling CBFM

While the PROMIS framework has usefully broadened perspectives on scaling, its breadth is also a weakness, for “it will not be feasible nor even desirable to apply the fully-fledged integrative perspective on each scaling initiative” (Wigboldus et al. 2016, p.45). The developers of PROMIS therefore suggest further refining of the framework to serve as a more relevant research tool (Wigboldus et al. 2016). The following section orientates a theory of scaling in the context of CBFM.

**Fig. 1:**
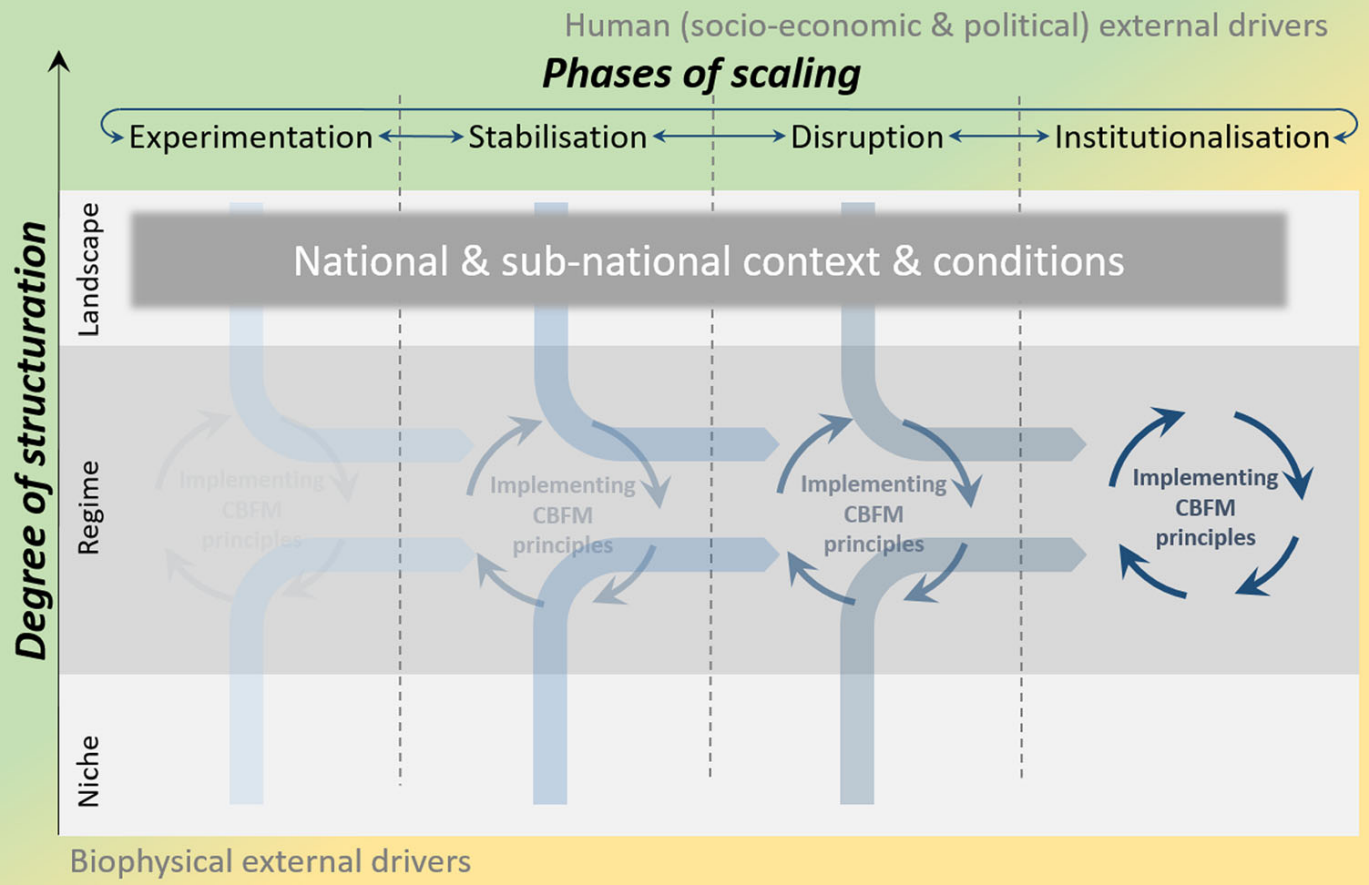
The theoretical scaffold of scaling CBFM, indicating the core conceptual elements that make up the framework for scaling CBFM shown in Fig. 2

**Fig. 2:**
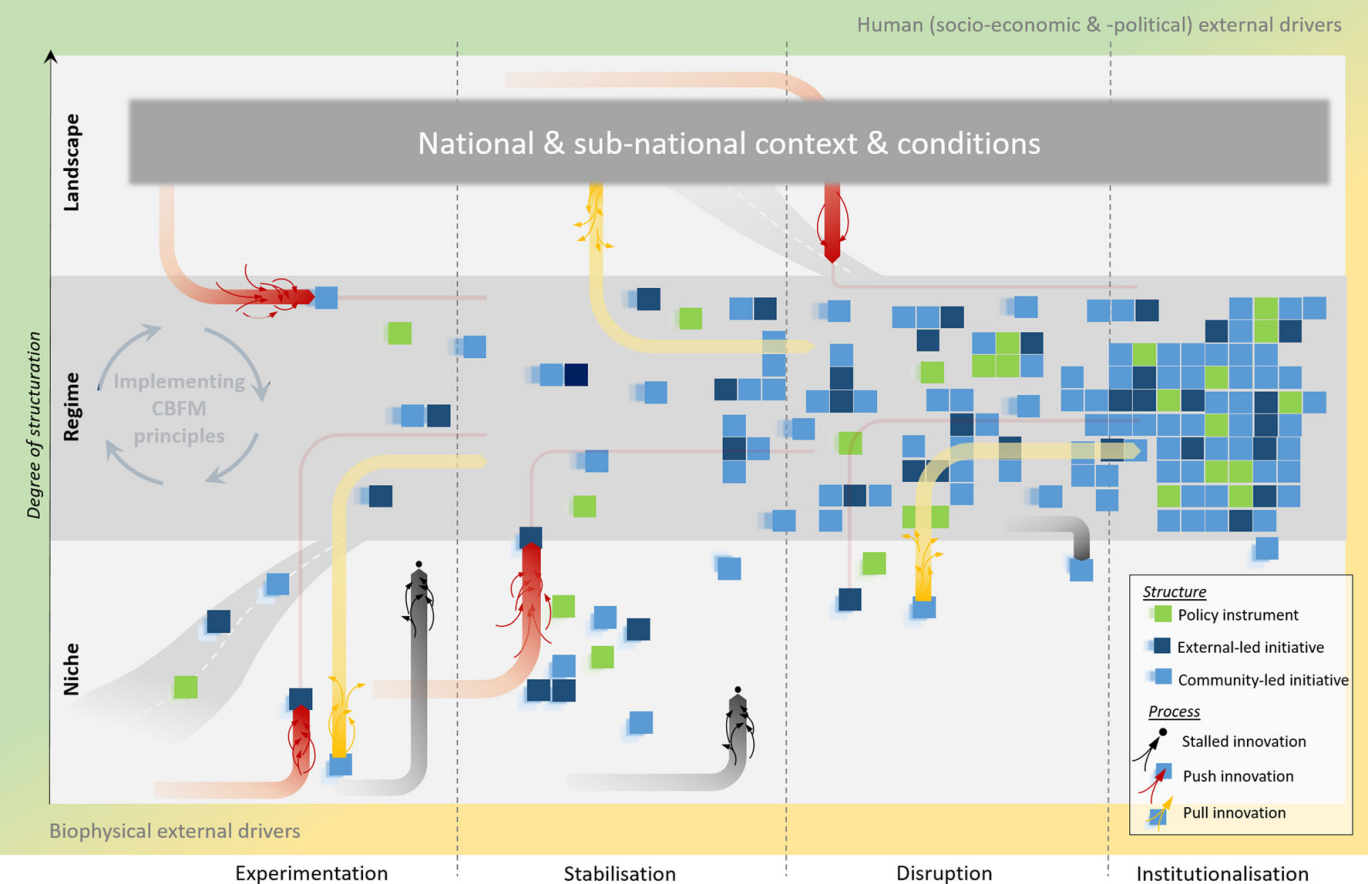
A conceptual framework for scaling CBFM that draws from PROMIS framing of scaling innovations. The figure depicts (spontaneous and deliberated) processes and structures transitioning a regime towards a desired outcome based on a generic vision of ‘implementing CBFM principles’; namely inter- and intra-connected sets of networks involving stakeholders doing and/or supporting CBFM that enables decentralized, polycentric governance of coastal fisheries over a defined large (national) space

The conceptual framework is illustrated in Figs. 1 and 2. Where Fig. 1 introduces the main elements of our conceptual framing around scaling CBFM, Fig. 2 builds on this by depicting the structural and process-driven changes involved in both direct (e.g. project interventions) and indirect (e.g. spontaneous adoption by communities) introductions of CBFM innovations. The framework presents a normative perspective on transition towards a desired situation; in this case widespread CBFM practice instead of reliance on centrally-governed fisheries management. Below we outline the different components of the framework.

### Distinguishing structure from process

Our framework makes explicit distinctions between structural and process-driven changes contributing to regime shifts towards widespread practice of CBFM. Structural change is characterized by the path-dependent sources of CBFM emergence, typically made visible through social behaviour or policy outputs supporting CBFM. Collective behaviour change in communities stemming from existing practices or local, independently-driven initiatives are represented by light blue blocks, while those resulting from externally-driven initiatives by projects or programs of NGOs, private sector or government agencies are represented by dark blue blocks (see Fig. 2). Similarly, supportive policies or amendments to national governing instruments that suit CBFM are represented by green blocks.

Process-driven change refers to the emergence of push and pull influences that work on structures. Push innovations are depicted by a series of red arrows converging on a particular structure, and may include national laws to which all citizens must adhere, or initiatives that respond to conflict or resource scarcity. Pull innovations are depicted by a series of yellow arrows fanning out from a particular structure, and may include national subsidy programs or non-government development funding opportunities. Black arrows converging on a single point represent stalled innovations; including trials that have ended in error or pilot initiatives that have not led to sustained uptake of any form. In the context of fisheries management, an example of a stalled innovation may include the disproportionate expansion of marine protected areas in relation to available management and enforcement capacity, resulting in ‘paper parks’ (De Santo 2013). Often, parts or lessons from stalled innovations may feed into new attempts at innovation, as, for example, when access rules around previously permanent fishery closure areas are adjusted spatially and/or temporally to address local food security and livelihood needs (Cohen and Steenbergen 2015).

The framework combines these processes and structures together conceptually. As structural CBFM results emerge from push and pull innovations, they create collective uptake trajectories, illustrated by increased smaller clumping of blocks from left to right in Fig. 1. With gradual but continued cumulative momentum in a relevant ‘direction’, a critical mass of uptake can contribute to regime shift, illustrated by dense clumping of blocks in a new configuration on the right in Fig. 2. We illustrate here several examples of depicted block-and-arrow combinations. Light blue blocks drawn upwards by yellow arrows, for example, may symbolize communities that independently practice resource management as part of local custom. Influences of pull innovations (yellow arrows), like social networks across communities that offer means to exchange learning, share management responsibilities and strengthen governance around fisheries, may see the community networks expanding as new communities subscribe to it (Nong and Marchke 2006). In doing so, customary practice finds compliment with broader strategies towards wider spread of management along coastlines. Dark blue blocks pushed upwards by red arrows, alternatively, may represent communities experiencing resource scarcity because of unsustainable fishing practices. The red arrows may be where the government has intervened to address this by, for example, establishing a collaborative arrangement to develop a local fisheries management plan. The deliberate interventions driving CBFM change encourage (i.e. push) a community towards fitting within a national coastal fisheries framework, thus simultaneously contributing to broader regime change. Green blocks representing national policy strategies that set out a development trajectory for coastal fisheries, or even global commitments (see sustainable development goals—UNDG 2015), may be framed as structural elements (exerting a push or inducing a pull) that drive local uptake and implementation of CBFM.

### Degrees of structuration

The framework adopts the scalar organization of Niche, Regime and Landscape that forms the foundation of socio-technical transition thinking, as developed by Geels (2002) for the MLP. These scales principally lay out degrees of structuration^1^ in practice, whereby relatively loose structural rigidity (“niche”) allows new innovations to develop more easily (but also disappear more easily), while gradually more rigid structure further up the scale (“regime” and “landscape”) provide more resistance to change (but also more permanent change once achieved).

The niche scale represents phases or spaces wherein new ideas and practices can be introduced and tested. These happen at small scales and with relative autonomy to the workings of the larger system. This allows ‘freedom’ from the restrictions of broader institutional norms, practices and/or rules that may otherwise restrain creative alternative approaches to be tested. This is not to say that a government’s broader institutional boundaries and rule systems do not apply. On the contrary, they define both a particular allowable operating space with limits to how far ‘pilot initiatives’ can deviate from the norm, and the basic ethical considerations around testing new ideas in communities. In CBFM context various approaches and instruments gain traction once proven to contribute to local empowerment, knowledge and capacity building. In most cases this involves harnessing existing local fishing knowledge, practices and institutions, as shown in the Pacific region where customary tenure systems form the foundation from which various co-management arrangements have sprung (Foale et al. 2011). In other cases, aspects of CBFM are introduced as something entirely new. In the Philippines, for example, community-based fishery monitoring activities has been introduced as a means to build knowledge of resource health and ecosystem behaviour, so as to inform adaptive management by a community and instil resource stewardship (Uychiaoco et al. 2005). Alternative livelihoods that make peoples’ dependence on natural resources more sustainable also frequent innovations as part of CBFM.

The regime scale represents “incumbent systems that involve dominant configurations relating to e.g. science, infrastructure, market and technology, and that have established ‘institutional logics’” (Funfschulling and Truffer 2014 In: Wigboldus et al. 2016, p.4). As a most basic interpretation, this scale represents ‘how things are done’ at any one time. At a national level, a country’s government operates according to principles and procedures enshrined in national constitutions that have developed from histories of civil engagement, social and political unrest, geopolitics, market influences and biophysical change. This makes regimes highly complex to interpret or depict in any one shape or form. With the continued influx of proven innovations over time, the regime may change in its configuration to a state that is more conducive to supporting CBFM.

As noted in previous sections the institutional make-up and logic of a regime means it functions in path dependent ways. This is depicted by way of merging pathways into the regime scale (see merging white dotted line in Fig. 1). Innovations may integrate and impact regime through such paths. Such paths are dynamic in their ability to take up new innovations. For example, strategic long-term planning instruments for national agencies to strengthen coastal fisheries may be developed at any one time based on existing policy, and in doing so pave way for new innovations that are in line with CBFM to be adopted in the future. Schwarz et al. (2020) outline how almost a decade’s worth of consultation in policy design processes in the Solomon Islands led to the eventual inclusion of CBFM plans in the Fisheries Act; thus formally recognizing customary marine tenure governance structures for the first time. They furthermore argue that its inclusion can empower local actors in fisheries management only if practitioners continually use it into the future. So where certain innovations can trigger more systemic change in the configuration and function of the regime, consistent adoption of these can lead then to more permanent transitions.

The landscape scale indicates the highest level of structure and exhibits strongest rigidity to change (Geels 2019). This refers to fundamental aspects of social and political reality in how people live. This is framed around culture, worldviews, politics and custom, and is shaped by biophysical character of the environment. Small island developing states (SIDS) with an atoll and archipelagic nature, for example, present a very different coastal fisheries landscape than that of a continental coastline. Similarly, places where traditional ecological knowledge and custom practices form the foundation of how people view and use fisheries will differ from places where globalizing influence of markets, migration, urbanisation and religion have become formative. Landscape scale influences can therefore inhibit or catalyse CBFM-related regime shifts.

### Phases of scaling

In outlining a theory of scaling CBFM, we incorporate the four phases of socio-technical transitions envisioned in the MLP (and PROMIS). In the first phase, niche-innovations gradually build up internal momentum through experimentation and trial-and-error. There would be a high degree of newness, uncertainty and chances of failure as well as competing claims and promises about CBFM. We may see a bourgeoning academic literature that attempts to apply the broad CBFM thinking into a specific locale, buoyed by new sources of funding supporting research in development initiatives (Shilomboleni and De Plaen 2019), and establishment of pilot sites. In the subsequent phase, CBFM would establish a foothold in the targeted geography. It stabilizes into a plausible large-scale design through activities such as experience sharing, standard setting and model building, which help to articulate best practices and how-to guidelines (Geels 2019). Often these are led by government fisheries departments and/or (external) development agencies. The articulation of positive cultural visions is also important in this phase to help legitimize the CBFM principles and attract wider backing (Song and Chuenpagdee 2014). Vocal opposition to CBFM may surface by social groups who experience negative side effects or feel insufficiently involved in decision making. At the same time, this is the phase where government officers, fishery extension workers, and fishing communities would start to embed or reinforce CBFM principles in the daily routines and practices (see Song et al. 2019).

The third phase, ‘disruption’, is characterized with the struggles between CBFM and the existing ways of managing fisheries (or lack thereof), as CBFM is pushed to take advantage of structural windows of opportunity created by landscape developments and niche-internal drivers. These struggles may play out in the economic domain involving fish capture and market institutions. The struggles could also engender policy disruptions as well as cultural and cognitive conflicts about the framing of fisheries problems and solutions. There is no guarantee that CBFM as niche-innovations will inevitably win these struggles. The principles of responsible scaling have told us that there are likely winners and losers from the process of scaling. Thus, disruptions, if anything, should generate an opportunity to critically reflect upon the merits of an emergent CBFM approach. If sufficient momentum is maintained, CBFM would then enter the fourth phase where it becomes institutionalized forming a ‘new norm’ that anchors regulations, fisher expectations and operational standards.

In reality, how CBFM is scaled to coastal communities is observed to follow a non-linear pattern, consistent with what we can expect in the non-orderly progression of these idealized phases (Geels and Raven 2006). Based on case studies in Solomon Islands, Abernethy et al. (2014) conclude that the studied communities moved forwards and backwards in their progression towards CBFM institutionalization based on the fluctuating degree of political support. Periods of rapid change or stagnation have also been documented. For example, CBFM in South Korea has so far failed to garner the sustained motivation of fishing communities despite the government’s vigorous promotion for nearly two decades commencing in the early 2000s (Park 2018). Understanding scaling via these four phases can therefore help raise crucial questions about the present and future trajectories of CBFM cases around the world.

### External drivers

Lastly, our framework recognizes the role of external drivers of change—both social and biophysical, as indicated in the outer perimeters of in Fig. 1—that can strengthen or destabilize niche and regime level developments, and landscape contexts. Economic external pressures such as macro-economic recessions or trade sanctions may erode the financial resource base of an otherwise thriving innovation, while socio-political external pressures may be related to social movement protests, changing government coalitions or persistent negative media coverage to affect the legitimacy and political support for an existing niche or regime innovation (Geels 2019). Community-based fisheries is prone to such external influences and more. High-level policy discourses and global finances have historically produced a sizable effect on the way community-based fisheries are imagined and practised, although not always in the best interest of the communities themselves (Steenbergen et al. 2017).

More sudden external influences include natural disasters and severe weather events, which can devastate coastal areas and overwhelm communities’ capacity to a quick recovery as learned from the typhoon impacts in the Philippines (Monteclaro et al. 2018). Most recently, the COVID-19 pandemic has critically exposed the vulnerability of coastal fisheries to the sudden realities of blocked market access, border restrictions and economic loss, creating a damaging effect on fish value chains and the livelihoods of the communities (FAO 2020). Scaling of CBFM in such extraordinary times, may potentially be undermined by emergency governing responses of the government taking precedence over community bylaws and other CBFM proceedings. Conversely, the (over)burdens caused by COVID-19 on central governments around the world can highlight the need for local fisheries management capacity as a means to overcome the pandemic-induced community vulnerabilities (Steenbergen et al. 2020). Such calls could add impetus among national and regional institutions towards furthering scaling progress.

## Conclusion

Despite increased focus on scaling local-level collective action institutions like CBFM, there has been less attention given to the theory guiding such initiatives and therefore an insufficient understanding of what it means to scale innovations of such type. Drawing on PROMIS thinking (Wigboldus et al. 2016) around agriculture innovations we move theory beyond diffusion perspectives, and assumptions of linear, sequential implementation, by presenting a framework with a more integrative, cumulative perspective that incorporates reflexive insights on what scaling CBFM might involve. This perspective takes into account process-driven as well as structural change across multiple levels of governance, phases of innovation and stages of institutionalisation. In doing so, we highlight the fundamental interplay between targeted action at the niche (e.g. community) scale and cumulative conditioning of an environment to enable change or drive ‘regime shifts’. In essence, we hypothesize that successful scaling requires engagement with all aspects of the governing regime, and therefore, is an enterprise that is larger than its parts.

The framework intends to lay the ground work for a comprehensive understanding of scaling CBFM. It provides a set of non-sequential areas of inquiry that direct us to (i) reflect on the potential positive and negative consequences of a scaling initiative (i.e. who benefits, who lags, are there ethical concerns, are there alternatives?); (ii) identify push and pull factors and their relative strengths and efficacy; (iii) understand regime-specific path-dependent ‘stickiness’ that may exert influence on scaling; (iv) examine the dynamics of scaling through combined process-driven and structural-driven changes; (v) assess, and potentially track, the relative ‘position’ of a scaling initiative in terms of degrees of structuration and phases of scaling; and (vi) consider impacts of external drivers on scaling trajectories. Such considered inquiry is imperative to allow subsequent translation into pragmatic tools and strategic guidance that can inform development of coherent (national) scaling programs. It can highlight potential entry points for design of effective monitoring mechanisms that drive mutually-responsive policy design and ground-level implementation. Box 1 briefly illustrates an application of the framework. Without offering an in-depth analysis, the CBFM case in Vanuatu preliminarily demonstrates the utility of the perspective offered through the four main dimensions of the framework (See “Distinguishing structure from process–External drivers” sections). Using the aforementioned ambition to develop ‘national CBFM scaling programs’ as an example of the way the framework can make sense of a regime shift over time, it highlights how the framework can further offer guidance and help to coordinate emerging CBFM scaling strategies and/or retrospectively identify gaps in scaling initiatives that are underway.

Although our thinking focuses in particular on scaling CBFM, the insights generated may be applicable across a broader set of initiatives that depend on local collective action for progress. Programs seeking spread in participatory natural resource management, rural livelihood enhancement, participatory conservation, remote health care provision and community development, all rely on synergetic collective organization at local levels. Ultimately, the framework holds potential as a tool with which to inform design and long-term planning for national development strategies. In that, the deliberate focus is on setting out principles and perspectives instead of proposing a type of governance model that would otherwise stipulate institutional structure and operational guidelines. Whereas the increased connectivity of a globalized world is often framed as a challenge to community development, sound theoretical guidance can harness such connectivity to strengthen networks of all relevant partners who make up nodes of collective action as part of national policy programs.

Box 1: An application of the theory for scaling CBFM Here we illustrate how the early evolution of a national coastal fisheries program can be usefully understood by considering a holistic scaling perspective as outlined by the different dimensions of the framework. In Vanuatu the evolution of CBFM can be traced to early initiatives in the 1990s. Raubani et al. (2017) write that at least seven externally-funded projects have had particular influence in shaping today’s approach to CBFM in Vanuatu. The aim to develop and refine a CBFM approach for broad application in Vanuatu was first demonstrated with the implementation of the Japanese government-funded project, “Grace of the Sea” in 2006. Later, ambitions to scale CBFM were made more explicit through the Australian Government-funded “Pathways” project. Important developments at all three scales of structuration have been at play. At the niche scale, the series of bilateral projects worked in communities in partnership with the government. The trochus rehabilitation programme in the early 1990s provided early lessons to the government about the importance of community engagement. Raubani et al (2017, p.8) make note of Johannes (1998)’s observation that the government “catalysed a striking upsurge in tradition-based marine resource management” among communities during that period. Niche innovations consistently followed, both in the government (e.g. see Sami et al. 2020) and non-government sector (e.g. see Neihapi et al. 2019), and over time were influential in the government’s recognition of CBFM in the national Fisheries Act. Further evidence of this is the long-term strategic planning framework that sets out a national vision to 2030 for CBFM (Vanuatu Fisheries Department 2019). At the regime scale, western governance influence over time established a foundation for centralised fisheries management. However, the various niche-level interventions over the years have helped the government to strongly adopt CBFM (Raubani et al. 2017). At the landscape scale, traditional structure and customary law provides the overarching cultural framework for regulating resource use and access, making the communities proforma owners of the resources.

CBFM outcomes often depend on internal community processes, not just the external support a community receives. According to Tavue et al. (2016), during the “Grace of the Sea” project, there was notable willingness of the communities to enrol in CBFM, which may be seen as the promising sign of CBFM spreading throughout Vanuatu. Even then, however, excessive reliance of CBFM systems on external agencies has been an ongoing issue. Once external support dries up, sustaining CBFM activities largely depends on the ability of communities to negotiate different visions within the communities, resolve conflicts and engineer ways to continue to enforce community rules (Léopold et al. 2013). The experiences of scaling CBFM in Vanuatu resemble the stage of stabilisation; through the continuing niche-level interventions in the form of external projects, combined with an increasingly strong commitment of the government, there is a plausible pathway to the adoption of a national program in Vanuatu. Influences of external drivers have been evident and will continue to impact pathways for change into the future. Various socio-political struggles have disrupted the trajectory, including pauses in foreign funding, introduction of new management paradigms, changes in government leadership or national emergency measures around COVID-19; as have biophysical disruptions in the form of natural disasters (tropical cyclone Pam and Harold in 2015 and 2020). All these elements play a part in constructing the trajectory of Vanuatu’s CBFM regime shift across scales of structuration and phases of innovation.
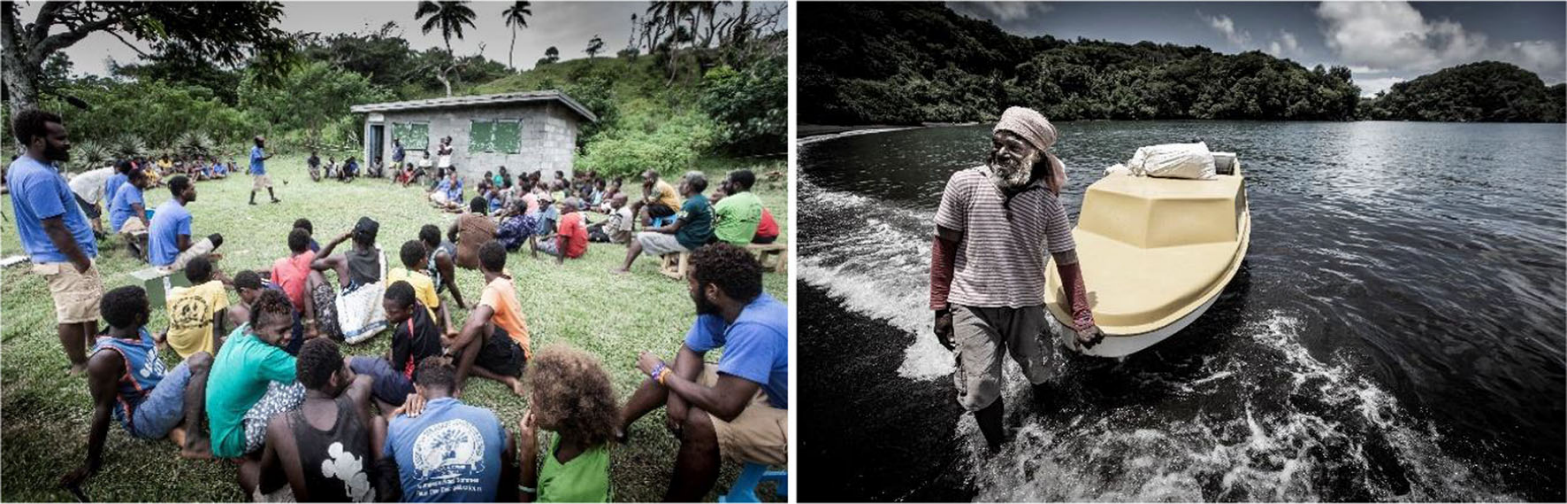

Photos: (i) CBFM workshop on Tanna Island, Vanuatu, and (ii) Small-scale fisher from Ambae Island, Vanuatu (Photos by Paul Jones)

## Footnotes

1 Adopting structuralist thinking from Giddins’ (1984) seminal work on organization and function of society, we interpret ‘structuration’ to mean the extent by which practices, norms and values in a system are ordered and stabilized within particular arrangements and rules.
